# Supplementary material for: Biological markers and psychosocial factors predict chronic pain conditions
Source: Nat Hum Behav. 2025 May 12;9(8):1710–25. doi: 10.1038/s41562-025-02156-y (PMC12367528; doi:10.1038/s41562-025-02156-y)
Supplement: Supplementary file 1 — Supplementary Methods and Figs. 1–11. [file 41562_2025_2156_MOESM1_ESM.pdf]

nature human behaviour

Supplementary information https://doi.org/10.1038/s41562-025-02156-y

Biological markers and psychosocial factors predict chronic pain conditions

In the format provided by the authors and unedited

Table of Contents

Supplementary Methods **................ 2**

Biological Modalities 2

Machine learning pipelines 7

Deviance explained calculation 8

Structure coefficients: 10

Supplementary Data Fig. 1: Blood assay structure coefficients for predictive models of diagnoses. 11

Supplementary Data Fig. 2: Blood assay weights for predictive models of diagnoses 12

Supplementary Data Fig. 3: Structure coefficients of bone structure features for predictive models of diagnoses. 13

Supplementary Data Fig. 4: Bone structure weights for predictive models of diagnoses. 14

Supplementary Data Fig. 5: Resting-state fMRI structure coefficients and weights for top 5 predictive models of diagnoses and widespread chronic pain 15

Supplementary Data Fig. 6: Diffusion and T1 imaging structure coefficients and weights for predictive models of diagnoses 16

Supplementary Data Fig. 7: Heritability estimates from Genome-Wise Association Studies (GWAS) of pain-associated diagnoses 17

Supplementary Data Fig. 8: Psychosocial structure coefficients for predictive models of diagnoses. 18

Supplementary Data Fig. 9: Psychosocial weights for predictive models of diagnoses. 19

Supplementary Data Fig. 10: Functional connectivity structure coefficients and weights for the Nociplastic Functional Signature (NFS). 20

Supplementary Data Fig. 11: Minimal impact of imputation method on model performance. 21

Supplementary Methods

Biological Modalities

Broad biological modalities indexing four diverse domains of physiological health were selected for analyses. Biological measures included blood immunoassays, polygenic risk scores, bone structure estimates derived from Dual-energy X-ray absorptiometry (DXA) scans, and brain imaging phenotypes. For certain analyses, we segmented blood and brain modalities into subcategories. Specifically, blood was divided into three distinct assay types: those assessing inflammatory or immune functions, metabolic functions, and hematological functions. Brain imaging was divided into: T1-weighted MRI for anatomical insights, diffusion MRI (dMRI) for white matter architecture, and resting-state functional MRI (fMRI) for brain connectivity.

The availability across study visits, derivation, and processing of each modality are detailed below:

Blood immunoassays (52 features, N = 476,787)

The UK Biobank measured 31 haematological (https://biobank.ctsu.ox.ac.uk/crystal/crystal/docs/haematology.pdf) and 30 biochemical (https://biobank.ndph.ox.ac.uk/showcase/ukb/docs/serum_biochemistry.pdf) blood assays using high fidelity haematology, immunoassay, and clinical chemistry analyzers. For the purposes of this study, 52 distinct assays were selected measuring a range of physiological functions including liver health (Gamma-Glutamyl transferase), kidney function (Cystatin C), systemic inflammation (C-Reactive protein), and immune activation (Leukocyte count), among others. Blood assays were collected twice: initially during the baseline evaluation of 502,494 participants and subsequently in a subset of 20,193 participants who returned approximately 4 years later (range: 1-6 years), hereafter referred to as the 4-year follow-up. At both time points, participants missing data for 22 or more assays (constituting over half of the selected assays) were excluded from further analysis. For the remaining participants with any missing data (up to 13% of the total sample, or a maximum of 62,216 individuals), we imputed missing values using the median of the available data. We also evaluated multiple Bayesian imputation, which produced concordant results (Supplementary Fig. 11). This process yielded a final analytic cohort of 476,787 individuals at baseline and 19,360 at the 4-year follow-up. To address distributional biases in raw assay measures, we applied logarithmic transformation to all immunoassay data prior to model training.

Polygenic Risk Scores (20 features, N = 408,597)

Blood samples collected at the baseline visit allowed different types of assays to be performed, including genetic data. A total of 488,118 underwent genotyping and have available phenotype information. Participants of similar genetic ancestry (Field: 22018), participants of non-European ancestry (Field: 22006), as well as participants who failed quality control (Field: 22010) were excluded from genetic analysis, resulting in 408,597 participants. This population was split into a training and a testing set, with the latter comprising individuals who attended the 9-year follow-up (n = 40,898), as conducted in Tanguay-Sabourin et al. 2023[6]. Consequently, polygenic risk scores were estimated within the training set of 367,699 participants and evaluated on the testing set of 40,898 participants as described below:

Genome Wide Association Studies (GWAS): For each training set across each pain phenotype and pain-associated diagnosis, we conducted genome-wide association studies (GWAS) using regenie[26]. Regenie was selected for its ability to address cryptic relatedness among UK Biobank (UKB) participants and manage case/control imbalances. To control for population structure in GWAS estimation, covariates were applied across both training and testing sets, including sex, age, age squared, genotyping array, the first 40 genetic principal components, and dummy-coded recruitment sites. Sex was determined based on genetically ascertained sex (XY=man, XX=woman; field 22001). In regenie's step 1, 93K SNPs identified by the UKB for kinship estimation were analyzed. For step 2 in regenie, approximately one million SNPs were evaluated, selected from the European-specific subset of the pan-UK Biobank project. These SNPs are high-quality HapMap 3 variants that meet five criteria: located in autosomes, outside the MHC region, bi-allelic, with INFO > 0.9, and MAF > 1% in both UKB and gnomAD datasets. Individuals retained for analysis were of European ancestry (field 22006), excluding any with failed genotyping quality, unusual heterozygosity, sex chromosome anomalies, or those who had withdrawn from the study by December 2021 (according to the sample-QC file from resource 531). Lastly, narrow-sense heritability estimates for each pain phenotype and diagnosis were derived using LDSC from the GWAS summary statistics[27].

Polygenic Risk Scores (PRS): Polygenic Risk Scores (PRS) were constructed ensuring that included SNPs were not in linkage disequilibrium, starting from the genotyping data in PLINK

format provided by the UK Biobank. Utilizing PLINK,[28,29] we removed all non-rsID SNPs as previously described, and excluded SNPs with MAF < 0.001, genotyping error rates > 1%, and Hardy-Weinberg equilibrium P-value < 10^{-12}. Linkage disequilibrium analysis among SNPs was performed within a 100 kb window, 100 variant steps, and an R^2 threshold of 0.2, resulting in 261,217 SNPs selected for PRS construction. These SNPs' GWAS effect sizes were aggregated to compute the PRS using PRSice-2 for each individual and pain phenotype, incorporating only SNP effect sizes from summary GWAS data of the training sets.[30] The PRS included all GWAS covariates and applied 20 P-value thresholds for SNP inclusion, ranging from P = $5x10^{-8}$ to P=1. Additionally, pathway-based PRS were analyzed using PRSet with Neural/Immune Gene Ontology (NIGO) pathways, focusing on those containing 10 to 100 genes for enhanced specificity.[31,32,33]

Brain imaging: A sub-sample of baseline participants were invited to attend a follow-up roughly 9 years later (range: 3-13 years), hereafter referred to as the 9-year follow-up. At the time of this study, data were available for 49,001 participants who had completed this follow up, which included a Magnetic Resonance Imaging (MRI) scan of the brain and the same questionnaires and assessments as baseline[3]. Structural and functional brain features were derived from three neuroimaging modalities, including T1-weighted MRI, diffusion MRI (dMRI) and resting-state functional MRI (fMRI). The image processing pipeline, artifact removal, cross-modality and cross-individual image alignment, quality control and phenotype calculation are described in detail in the central UK Biobank brain imaging documentation (https://biobank.ctsu.ox.ac.uk/showcase/showcase/docs/brain_mri.pdf) and by Alfaro-Almagro and colleagues[4]. Additionally, we excluded participants with excessive head motion or suboptimal signal-to-noise ratio, as well as those whose T1 brain images required excessive warping for non-linear alignment to standard space, defined quantitatively as deviations greater than three standard deviations from the group mean (exceeding the 99.75th percentile).

T1-weighted MRI: (1041 features, N = 41,979) T1-weighted MRI features include regional and subcortical gray matter volume, cortical thickness, surface area, and volume, regional gray/white matter intensity contrast as derived from T1-weighted MRI. Participants missing data for more

than 75% of the features (exceeding 780 features) were excluded from further analysis, the remaining missing data was imputed using the median.

Diffusion-weighted MRI: (614 features, N = 36,779) Diffusion-weighted MRI features include microstructural measures of white matter tracts including mean fractional anisotropy and mean diffusivity as well as derivatives of diffusion tensor imaging including orbital diffusivity, MO, L1, L2, ICVF, and ISOVF. Participants missing more than 75% of the features (exceeding 461 features) were excluded from further analysis. This resulted in the final sample of 36,779 individuals with complete diffusion features, as such no imputation was required.

Resting state functional MRI: (38,781 features, N = 37,414) Resting-state functional Magnetic Resonance Imaging data is based on the minimally preprocessed pipeline designed and carried out by the FMRIB group, Oxford University, United Kingdom. The minimally preprocessed resting-state fMRI data from the UK Biobank were analyzed using the following preprocessing steps: motion correction with MCFLIRT[4], grand-mean intensity normalization, high pass temporal filter, fieldmap unwarping, and gradient distortion correction. Noise terms were identified and removed using FSL ICA-FIX. Full information on the UK Biobank preprocessing is published[4]. Additional preprocessing included warping the image in native space to the 3mm MNI template (FSL), despiking using 3DDespike (AFNI from Nipype), 6-mm kernel smoothing (Nilearn), and resampling to 3-mm (for storage purposes). A modified Brainnetome atlas[5] including additional midbrain, brainstem, and cerebellar regions was used to parcel the brain into 279 distinct regions. Dynamic connectivity was estimated between each region to derive a functional connectome using Dynamic Conditional Correlation (DCC). DCC is based on generalized autoregressive condition heteroscedastic (GARCH) and exponential weighted moving average (EWMA) models (implemented by https://cocoanlab.github.io/tops/).

Bone structure: (68 features, N = 40,815) At the time of this study, measures from Dual-energy X-ray Absorptiometry (DXA) bone scans were available for 40,815 participants at the 9-year follow-up (https://biobank.ndph.ox.ac.uk/showcase/ukb/docs/DXA_explan_doc.pdf). Radiographers analyzed the scans at the whole body, spine, and hip levels either during or shortly after the image acquisition. This analysis facilitated the generation of comprehensive numerical

measures, including bone area, bone mineral content (BMC), and bone mineral density (BMD). A total 91 features across seven bone systems—spine, femur, head, legs, pelvis, arms, and ribs were extracted by the UK Biobank. From this pool, we excluded features with low coverage (fewer than 10,000 participants or 25% of the sample), narrowing the feature space to 68. Among the 40,815 participants assessed, we imputed any remaining missing data, which affected 4.9% or 2,015 values, using the median.

Psychosocial Phenotypes (81 features, N = 493,211)

We used a truncated set of psychosocial features originally defined in our recent paper, see Tanguay-Sabourin, C. *et al* for details[6]. As the goal of the present study is to compare biological predictors to psychosocial predictors, we excluded biological features included in the original model to generate a strictly psychosocial modality, including BMI, age, sex, ethnicity, grip strength, and blood pressure. Thus, a total of 81 features collected at the baseline and follow-up visits were selected a-priori based on their relevance to chronic pain. The selection was based on the Prognosis Research Strategy (PROGRESS) group who recently provided a framework for the development of a prognostic model to determine risk profile[7]. Variables were organized through an iterative approach along a hierarchical framework from 81 features into 8 categories forming three distinct domains (i.e., mental health, physical health, and sociodemographic). The three domains are as follows:

Mental health: The mental health domain includes 3 categories: i) neuroticism – all individual items and their total sum-score – based on 12 neurotic behaviors closely linked to negative affect, ii) traumas – illness, injury, bereavement, or stress in the last 2 years – including 6 events, and iii) mood – reported frequency of certain moods in the past 2 weeks and visits to a general practitioner or psychiatrist for nerves, anxiety, tension, or depression.

Physical health: The physical health domain includes 3 categories: i) physical activity – Metabolic Equivalent Task (MET) scores computed using the International Physical Activity Questionnaire (IPAQ) guidelines[8], ii) sleep – questions regarding duration, napping, snoring, and sleeplessness, iii) substance use – smoking and alcohol use.

Sociodemographic: The sociodemographic domain includes 2 categories: i) socioeconomic status – education completed, income, employment, etc., ii) occupational measures – individuals present within household, social entourage, and job type (e.g., manual or physical job).

For a detailed view of the biological and psychosocial features included in this study, see **Supplementary Tables 1-6**.

Machine learning pipelines

Blood biochemistry pipeline: A significant portion of participants within the diagnosis groups reported taking medications known to alter blood biochemistry. These include, but are not limited to, hypertensive drugs, anti-rheumatic medications, and immunosuppressants. Recognizing the potential biases these medications could introduce in the biochemical features under study, we incorporated propensity score matching to control for them. This approach aimed to align subjects from the disease groups with their counterparts in the control group based on self-reported medication intake.

While unable to eliminate all confounding effects of medications, our method effectively mitigated a considerable extent of the confounding impact, as evident from **Extended Data Fig. 6**. To provide a brief overview:

- **Propensity Score Estimation**: Propensity scores, which represent the likelihood of an individual being treated considering specific confounds, were generated via a logistic regression model. We extracted confounds by implementing the top 5 components from a mixed factor analysis on the predominant 5 medications, including statins, associated with each disease group. This, combined with age and sex, served as the foundation for our matching process between case and control groups.

- **Matching Process**: Each individual from the case group was paired with a counterpart in the control group using the Hungarian matching algorithm[14]. This algorithm determines an optimal match by minimizing the overall distance or "cost" between paired subjects. This distance is calculated based on the absolute difference in their estimated propensity scores. A conservative caliper, defined as 0.2[11], ensures that this difference does not surpass a predetermined threshold.

- **Timing and Application**: It's important to note that propensity score matching was executed prior to the cross-validation data split. This ensured consistency in the data across every modeling iteration.

Polygenic risk score pipeline: In the polygenic risk score models, we applied a thresholding procedure to the outputs of each diagnosis GWAS, yielding 20 levels of statistical significance thresholds for each single nucleotide polymorphism, ranging from P = $5x10^{-8}$ to P=1. Models were subsequently trained on these thresholds to determine the optimal P-value threshold to distinguish between the pain outcome group and the healthy control group, without adjusting for any confounding variables.

Brain imaging pipeline: Brain imaging models were trained on multi-modal brain imaging features including white matter features from diffusion-weighted imaging, structural features from T1-weighted imaging, and functional connectivity features from resting state imaging. Several confounding variables were adjusted for in brain imaging pipelines: average absolute head motion, volumetric scaling (to account for variations in brain size) from the T1 image to a standardized space, age, and sex.

Bone structure pipeline: Bone structure models were trained on DXA derived features including bone area, bone mineral content (BMC), and bone mineral density (BMD), and were adjusted using age and sex as confounding variables.

Psychosocial pipeline: To align with the methodologies employed in other modalities we adjusted for age and sex as confounding variables in our models that were trained on psychosocial features.

Deviance explained calculation

The deviance explained by biological factors, psychosocial factors, and their unions in classifying two distinct chronic pain conditions - rheumatoid arthritis and fibromyalgia - was assessed using the optimal biological modalities for each condition, determined by the highest average AUC scores: blood for rheumatoid arthritis and brain for fibromyalgia. These calculations were then compared with the deviance explained in models of self-reported chronic pain, employing the same biological modalities for comparability.

The deviance explained (D^2) by the biological (B), psychosocial (P), and combined (B+P) predictors was calculated following a modified approach based on the methodology outlined by

Dinga et al. 2020, adapted to our context of comparing different types of predictive information rather than controlling for confounds[12]. First, predicted probabilities were extracted from models trained on: biological predictions (B) from the blood or brain models, psychosocial predictions from the models trained on psychosocial data (P), and combined data (B+P) integrating both biological and psychosocial predictions.

Logistic regression models were then fit on these predicted probabilities to predict each specified outcome (either the diagnosis or self-reported chronic pain), generating:

- A model using only predicted probabilities from biological data (B).
- A model using only predicted probabilities from psychosocial data (P).
- A model using combined predicted probabilities from both data sources (B+P).

The deviance for each predictive model was then calculated using the following formula:

$$\text{Deviance} = 2 \times (\text{loglikelihood}_{\text{saturated}} - \text{loglikelihood}_{\text{model}})$$

Where the saturated model represents a model with perfect fit to the data and the maximum possible log-likelihood. Subsequently, the fraction of deviance explained (D^2) for each model was derived by normalizing the model's deviance against that of a null model, which includes only an intercept, according to:

$$D^2 = 1 - \frac{\text{Deviance}_{\text{model}}}{\text{Deviance}_{\text{null}}}$$

To dissect the deviance explained into unique and shared contributions, the following calculations were performed:

- $\Delta D^2_{\text{P}} = D^2_{\text{BP}} - D^2_{\text{B}}$: The unique contribution of psychosocial predictions beyond what is explained by biological predictions.
- $\Delta D^2_{\text{B}} = D^2_{\text{BP}} - D^2_{\text{P}}$: The unique contribution of biological predictions beyond what is explained by psychosocial predictions.
- $\Delta D^2_{\text{B}_\cup\text{P}} = D^2_{\text{P+B}} - \Delta D^2_{\text{P}} - \Delta D^2_{\text{B}}$: The shared or overlapping contribution of biological and psychosocial predictions to the explained deviance.

Where D^2_{BP}, D^2_{B} , D^2_{P} are deviance explained of models containing biological and psychosocial predictions, biological predictions, and psychosocial predictions, respectively. These results quantify the extent to which biological and psychosocial factors individually and jointly explain the variance in the binary disease or self-reported pain outcome and are displayed in **Fig. 1D**.

Structure coefficients

Given the potential instability and bias in feature weights derived from multivariate predictive models, we utilized structure coefficients, or model encoding maps, to identify features individually linked to the outputs of our predictive machine learning models[20]. Structure coefficients, recognized in brain imaging and predictive modeling research[21,22], provide a means to extract reliable features associated with a predicted outcome. Specifically, these coefficients reveal the individual association of features—in this case the Dynamic Conditional Connectivity (DCC) between two brain regions of interest (ROIs)—with the model's output—in this case the predicted probability of a nociplastic pain condition—thereby mapping individual features to the overall multivariate model prediction.

For each participant, structure coefficient maps were generated by computing the covariance between the predicted probabilities from the logistic regression model and the parcel-wise DCC values. This process created an encoding map delineating the directionality of the relationship between each feature and the model prediction. In our analysis, this method maps which brain voxels were positively or negatively associated with the predicted nociplastic condition. For all predictive models, structure coefficient maps were calculated for each of the five iterations of the machine learning pipeline and subsequently averaged.

A | Blood assay structure coefficients for pain-associated diagnoses

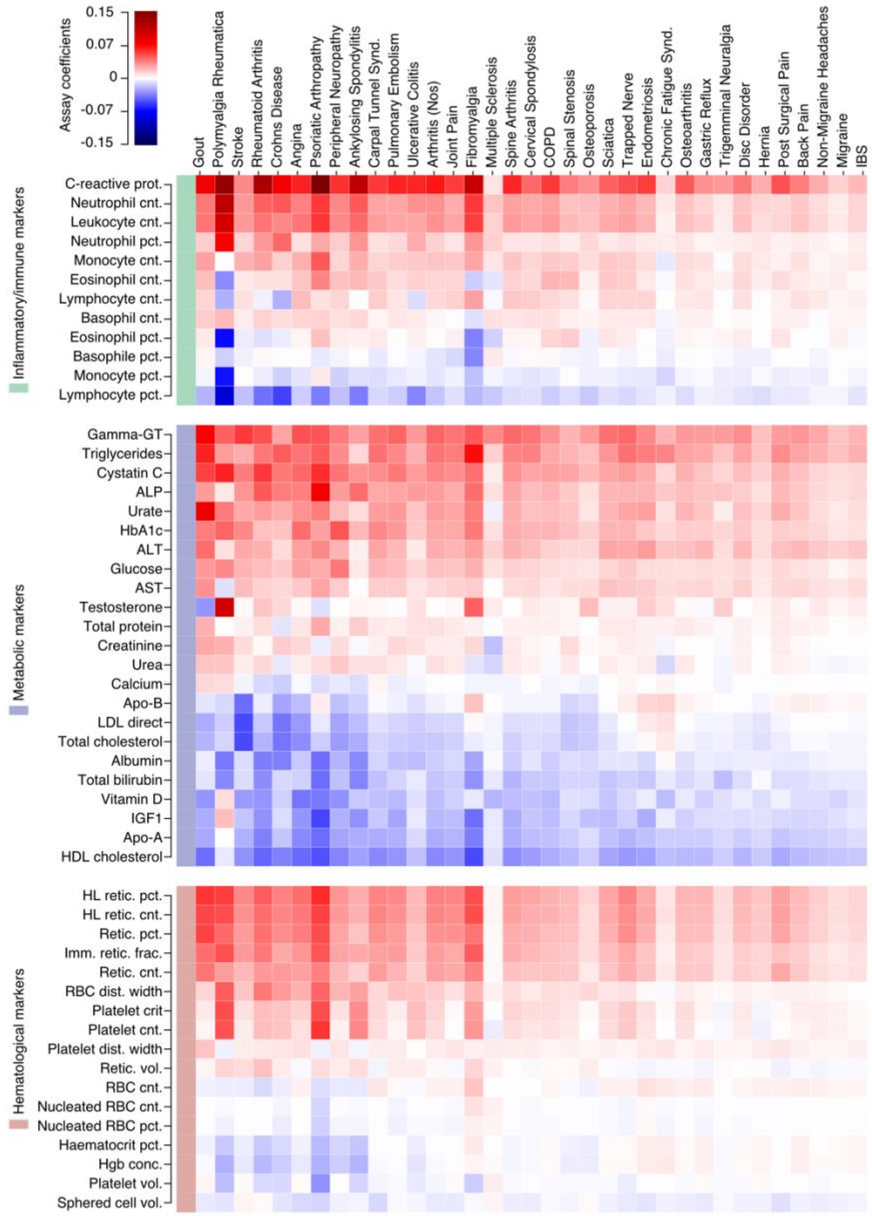

Supplementary Data Fig. 1: Blood assay structure coefficients for predictive models of diagnoses. Structure coefficients from logistic regression models, which were trained on blood assay data to predict pain-associated diagnoses, are displayed for each diagnosis. They are organized in descending order of their predictive accuracy (ROC-AUC) for each respective diagnosis and segmented into subcategories of inflammatory/immune markers, metabolic markers, or hematological markers. ALP, alkaline phosphatase; HbA1c, glycated hemoglobin, Glu, glucose; ALT, alanine aminotransferase; AST, aspartate aminotransferase; ApoB, apolipoprotein b; IGF1, insulin-like growth factor; LDL-C, low density lipoprotein cholesterol; HL, high light scatter; Retic, reticulocyte; Imm. retic. frac., immature reticulocyte fraction; PDW, platelet distribution width; RBC; red blood cell.

A | Blood assay weights for pain-associated diagnoses

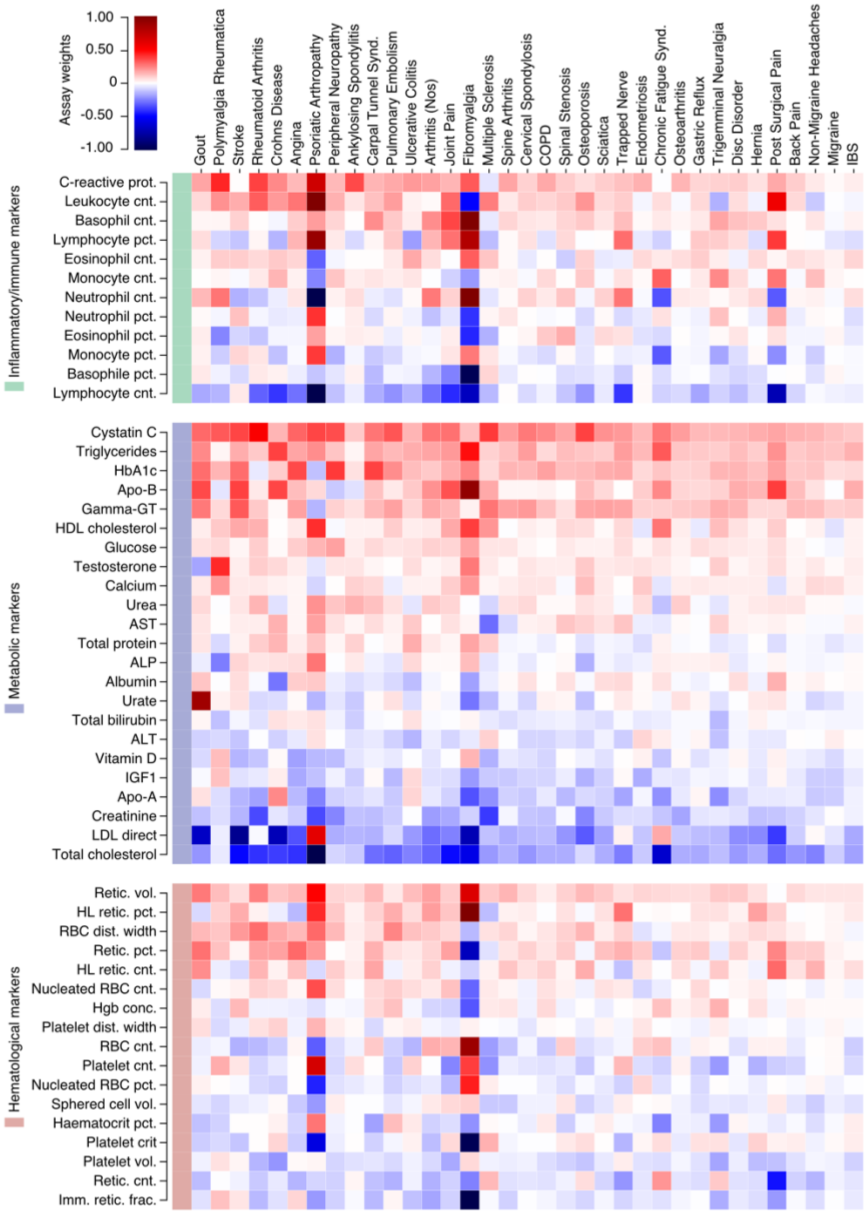

Supplementary Data Fig. 2: Blood assay weights for predictive models of diagnoses. Feature weights from logistic regression models, which were trained on blood assay data to predict pain-associated diagnoses, are displayed for each diagnosis. They are organized in descending order of their predictive accuracy (ROC-AUC) for each respective diagnosis and segmented into subcategories of inflammatory/immune markers, metabolic markers, or hematological markers. ALP, alkaline phosphatase; HbA1c, glycated hemoglobin, Glu, glucose; ALT, alanine aminotransferase; AST, aspartate aminotransferase; ApoB, apolipoprotein b; IGF1, insulin-like growth factor; LDL-C, low density lipoprotein cholesterol; HL, high light scatter; Retic, reticulocyte; Imm. retic. frac., immature reticulocyte fraction; PDW, platelet distribution width; RBC; red blood cell.

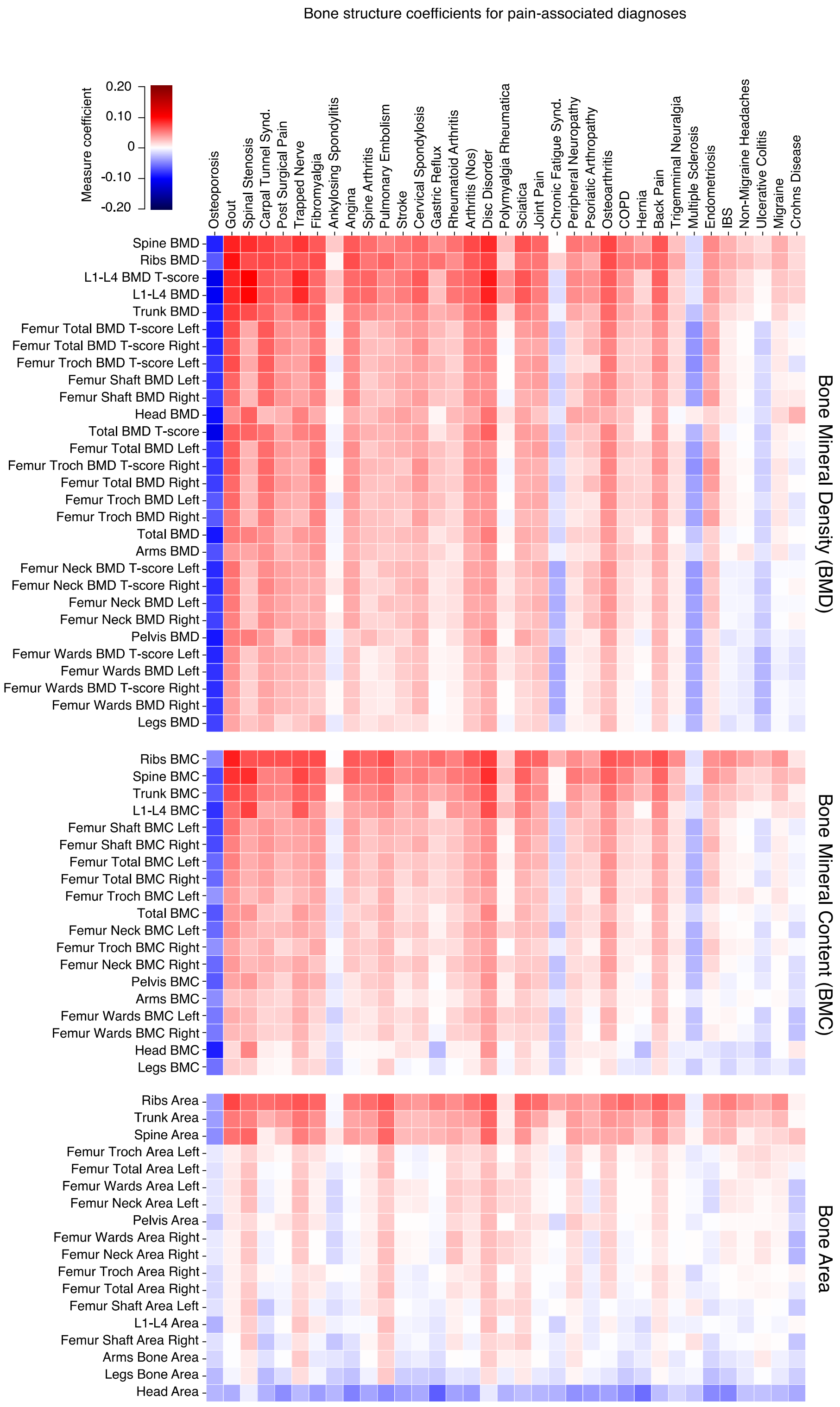

Supplementary Data Fig. 3: Structure coefficients of bone structure features for predictive models of diagnoses. Structure coefficients from logistic regression models, which were trained on Dual X-ray Absorptiometry (DXA) scan data to predict pain-associated diagnoses, are displayed. They are organized in descending order of their predictive accuracy (ROC-AUC) for each respective diagnosis.

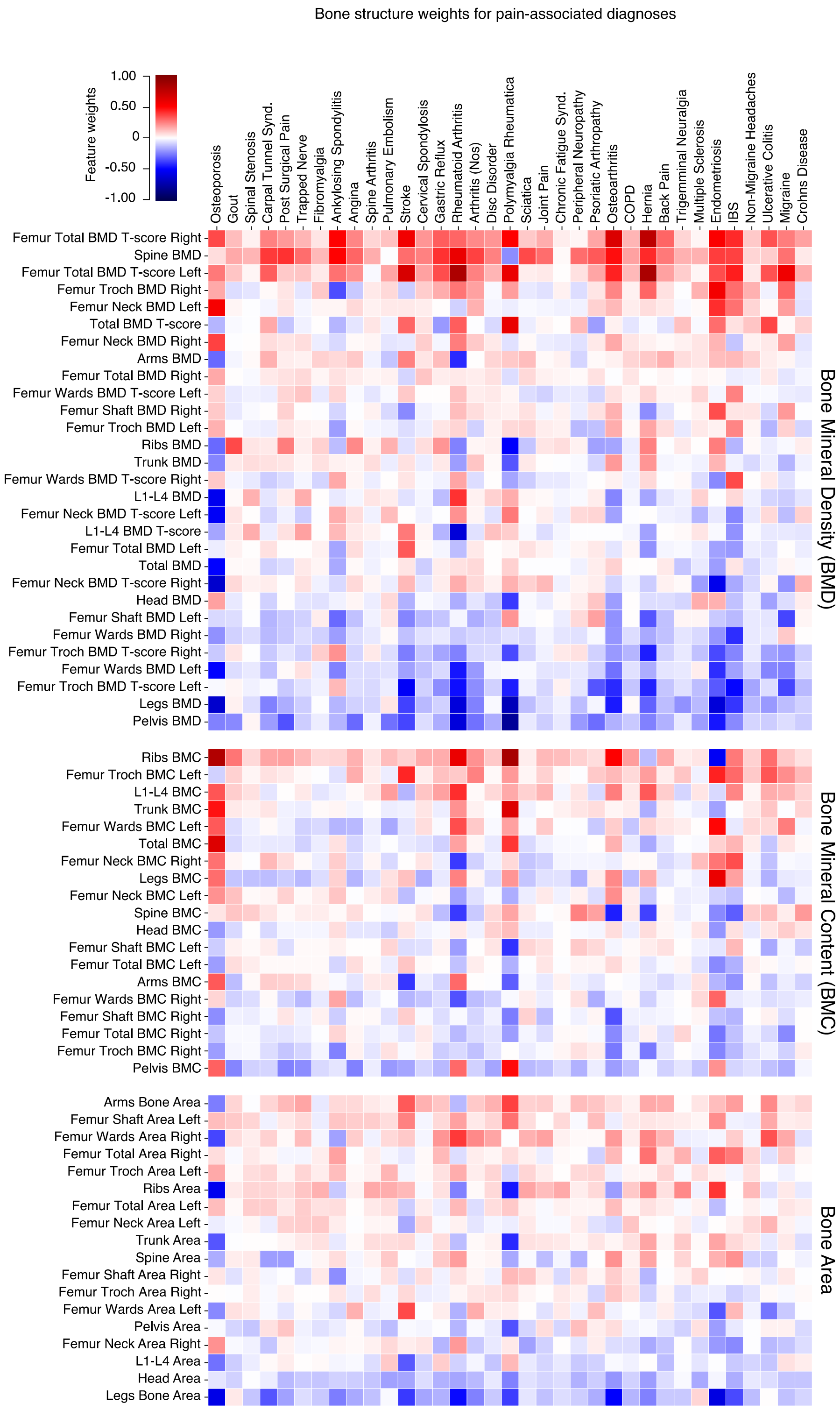

Supplementary Data Fig. 4: Bone structure weights for predictive models of diagnoses. Feature weights from logistic regression models, which were trained on Dual X-ray Absorptiometry (DXA) scan data to predict pain-associated diagnoses, are displayed. They are organized in descending order of their predictive accuracy (ROC-AUC) for each respective diagnosis.

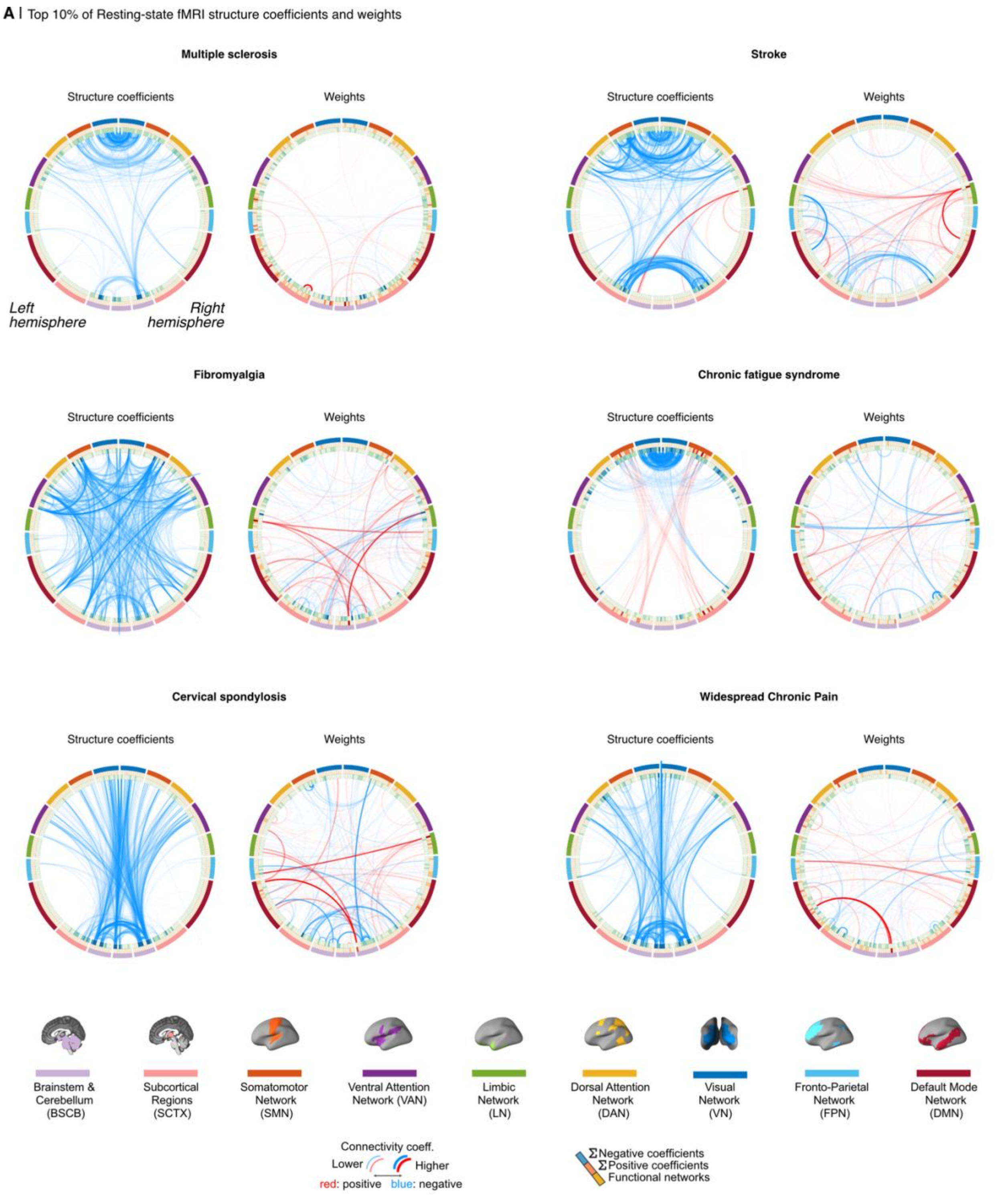

Supplementary Data Fig. 5: Resting-state fMRI structure coefficients and weights for top 5 predictive models of diagnoses and widespread chronic pain. Dynamic conditional correlation (DCC) functional connectivity structure coefficients and weights for brainnetome atlas parcels were derived from logistic regression models trained on resting-state fMRI features to classify pain-related diagnoses. The top five diagnoses with the highest ROC-AUC scores are highlighted, along with widespread chronic pain. In this context, coefficients indicate the unique impact of parcel-to-parcel connectivity on diagnostic predictions, while weights show the predictive strength and direction, adjusted for other model connections. Circos plots visualize the top 10% of influential connections as links between distinct parcels, categorized by resting-state network.

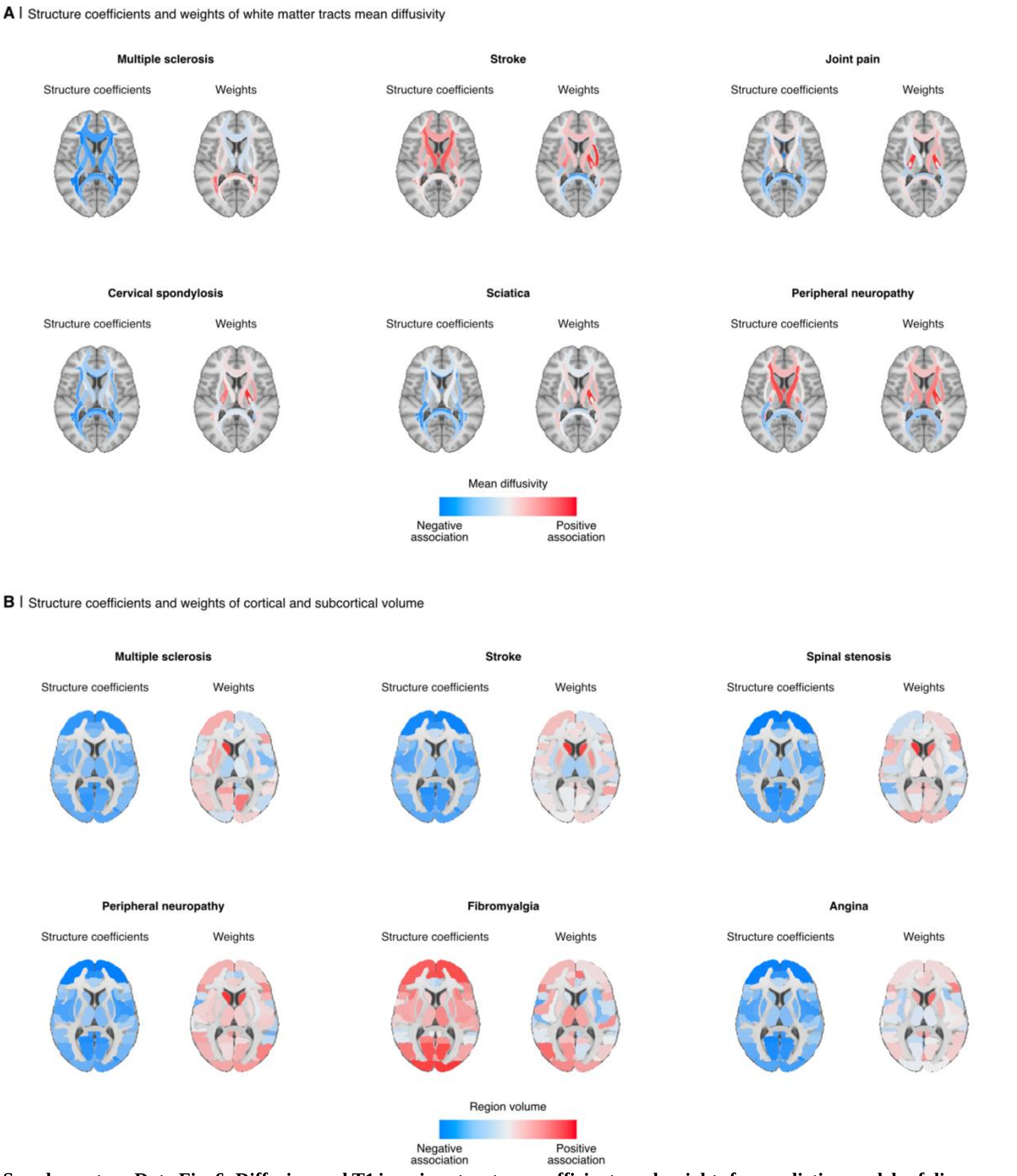

Supplementary Data Fig. 6: Diffusion and T1 imaging structure coefficients and weights for predictive models of diagnoses. A. Mean diffusivity structure coefficients and weights from white matter tracts were derived from logistic regression models using diffusion-weighted imaging features to classify pain-associated diagnoses. The six diagnoses yielding the highest ROC-AUC scores are presented, with their respective coefficient/weight values representing the impact of mean diffusivity within each tract on the predicted diagnosis. These values are mapped onto a white matter tract atlas superimposed on an MNI152 brain template. **B.** Similarly, structure coefficients and weights for cortical and subcortical volumes are displayed for the six diagnoses most accurately predicted using T1 structural brain imaging features. It is important to note that the features shown here represent a subset for clarity and do not encompass all the features utilized in the machine learning models. Specifically, mean diffusivity from diffusion weighted imaging and regional volumes from T1 imaging are highlighted for illustrative simplicity.

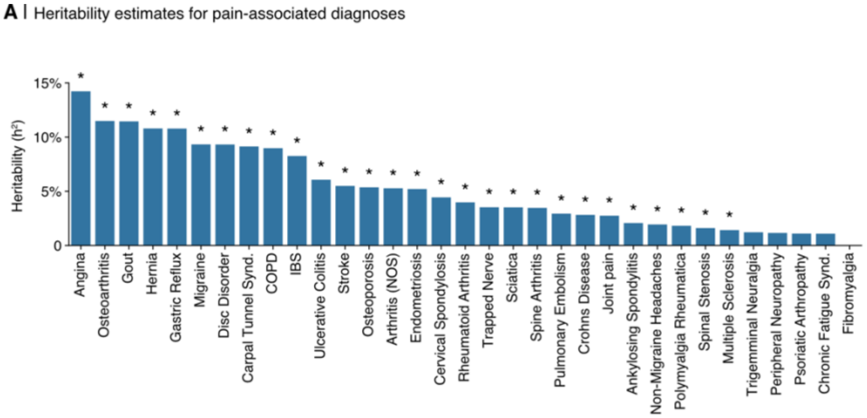

Supplementary Data Fig. 7: Heritability estimates from Genome-Wise Association Studies (GWAS) of pain-associated diagnoses. Narrow-sense heritability estimates obtained via LDSC (Linkage Disequilibrium Score Regression) from GWAS summary statistics are provided for each pain-associated diagnosis. Statistical significance is determined by two-sided, FDR-corrected Wald Test with P values less than 0.05. All $P_{\mathrm{FDR}} < 0.001$, except for polymyalgia rheumatica ($P_{\mathrm{FDR}} = 0.022$), spinal stenosis ($P_{\mathrm{FDR}} = 0.016$), multiple sclerosis ($P_{\mathrm{FDR}} = 0.044$), trigeminal neuralgia ($P_{\mathrm{FDR}} = 0.092$), peripheral neuropathy ($P_{\mathrm{FDR}} = 0.086$), psoriatic arthropathy ($P_{\mathrm{FDR}} = 0.093$), chronic fatigue syndrome ($P_{\mathrm{FDR}} = 0.105$), and fibromyalgia ($P_{\mathrm{FDR}} = 0.951$).

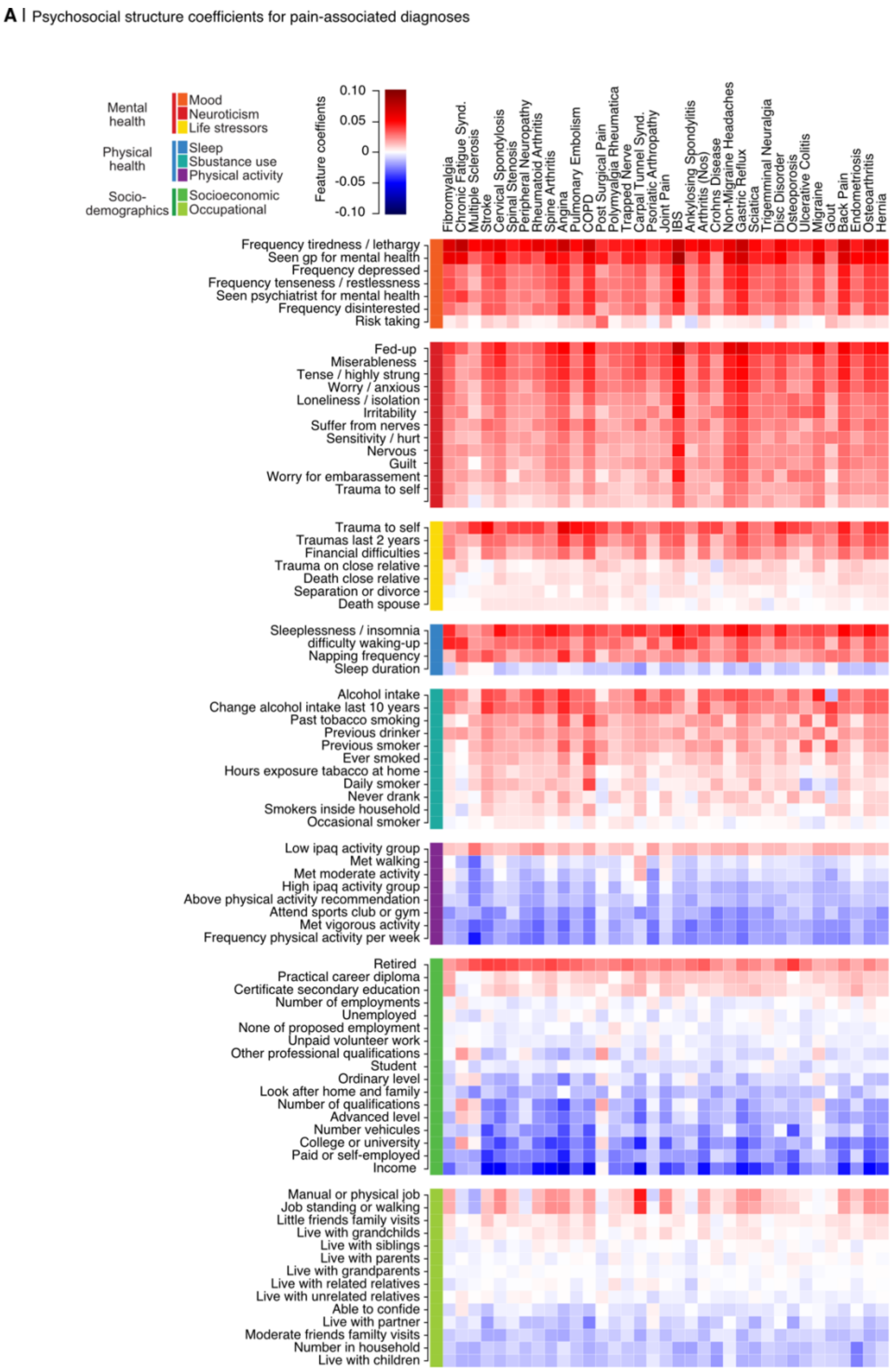

Supplementary Data Fig. 8: Psychosocial structure coefficients for predictive models of diagnoses. Feature weights from logistic regression models, which were trained on psychosocial variables to predict pain-associated diagnoses, are displayed for each diagnosis. They are organized in descending order of their predictive accuracy (ROC-AUC) for each respective diagnosis. IPAQ, International Physical Activity Questionnaire; MET, metabolic equivalent task.

A | Psychosocial weights for pain-associated diagnoses

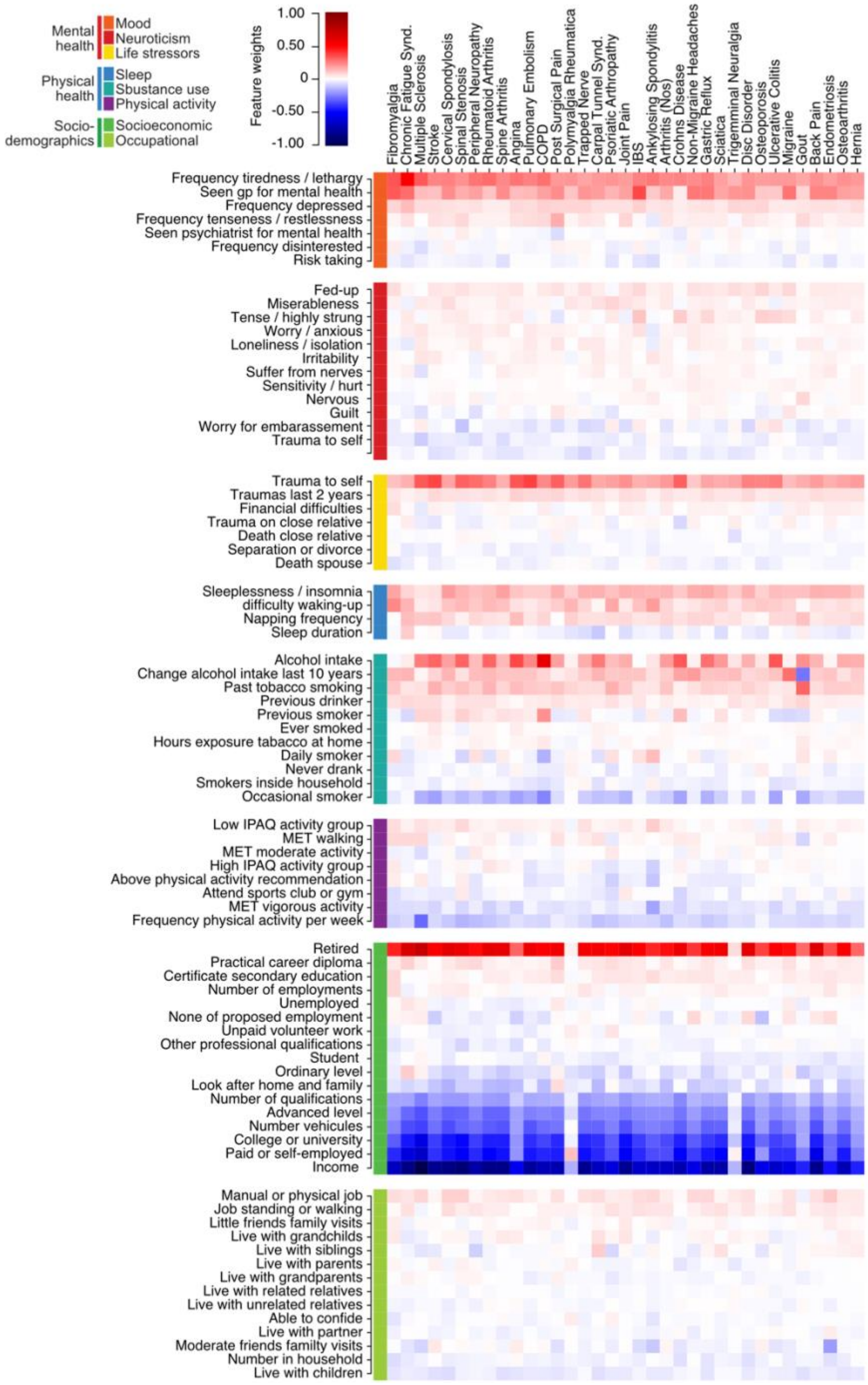

Supplementary Data Fig. 9: Psychosocial weights for predictive models of diagnoses. Feature weights from logistic regression models, which were trained on psychosocial variables to predict pain-associated diagnoses, are displayed for each diagnosis. They are organized in descending order of their predictive accuracy (ROC-AUC) for each respective diagnosis. IPAQ, International Physical Activity Questionnaire; MET, metabolic equivalent task.

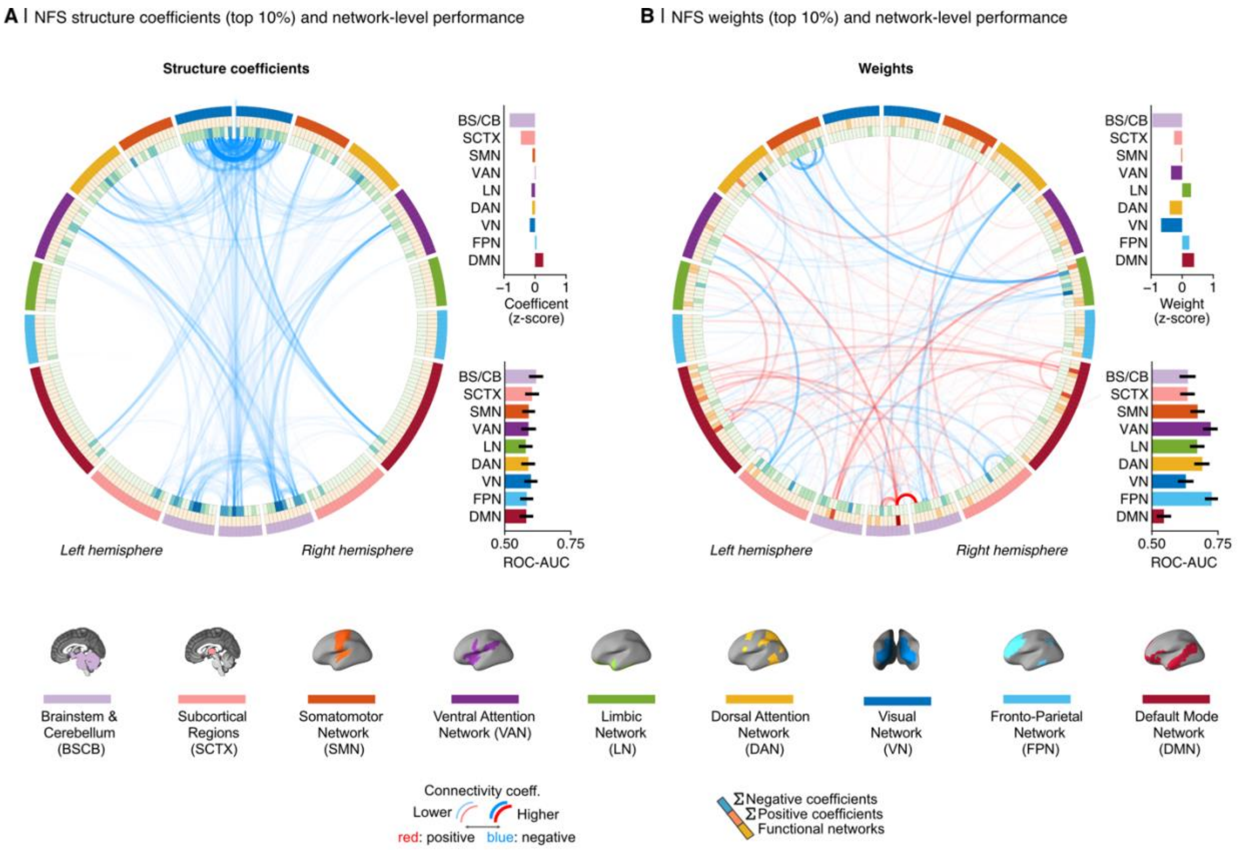

Supplementary Data Fig. 10: Functional connectivity structure coefficients and weights for the Nociplastic Functional Signature (NFS). A. Left: Circos plot illustrating the top 10% of connectivity links, as identified by structure coefficients from the NFS resting-state fMRI model, mapped across the canonical resting-state networks. Each cell represents a brain region of interest colored based on the density of positive (in red) and negative (in blue) edges. Top right: Average structure coefficient for edges within each resting state network, with values normalized across all edges. Bottom right: Classification performance (ROC-AUC) of edges within each resting state network for distinguishing nociplastic conditions from diagnosis-free controls, based on coefficients from the full model. Error bars show 95% CI from 1,000 bootstrap samples based on an original sample size of n = 4780. **B.** Same as A but using the logistic regression model weights instead of structure coefficients. Error bars show 95% CI from 1,000 bootstrap samples. Abbreviations: Brainstem and Cerebellum (BSCB); Somatosensory Network (SMN); Subcortex (SCTX); Ventral attention Network (VAN); Limbic Network (LN); Dorsal Attention Network (DAN); Visual Network (VN); Fronto-Parietal Network (FPN); Default Mode Network (DMN).

A

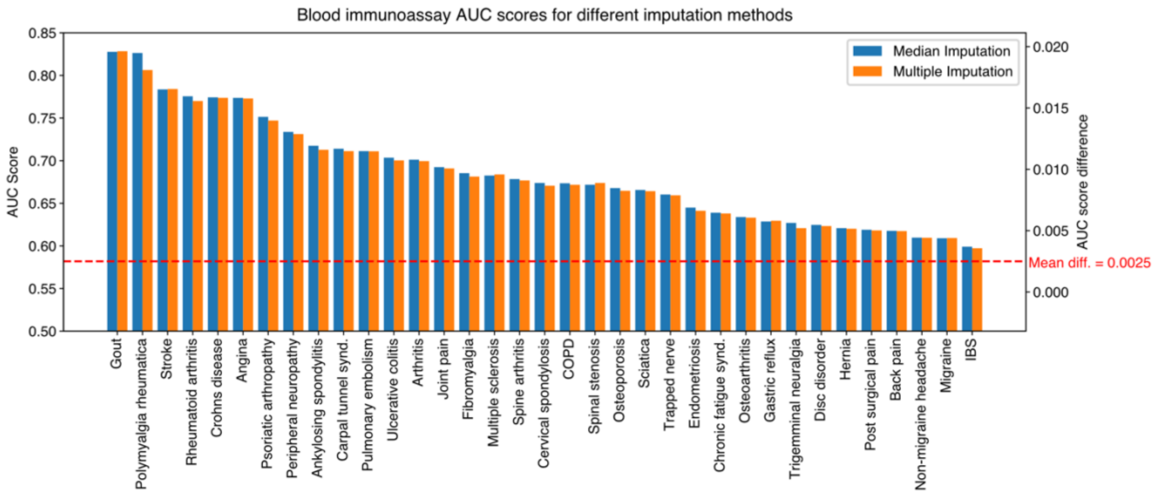

B

Differences in mode performance metrics between imputation methods
(Median imputation score - Multiple imputation score)

	Log accuracy	Brier score	Pseudo R2
Mean difference	-0.0002	-0.0006	0.0007
Max difference	0.0477	0.0051	0.0211
Min difference	-0.0252	-0.0098	-0.0488

C

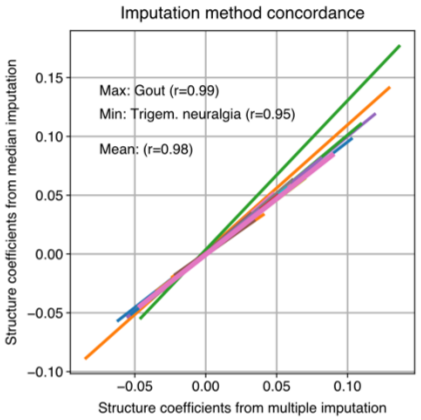

Supplementary Data Fig. 11: Minimal impact of imputation method on model performance. A, Comparison of AUC scores from a machine learning models using two different imputation techniques for handling missing blood immunoassay data: median imputation and multiple Bayesian imputation. **B,** Extends the comparison in (A) to additional performance metrics: logarithmic accuracy, Brier score, and pseudo R-squared, illustrating the robustness of model outputs under different imputation strategies. **C,** Plots the relationship between structure coefficients (SC) derived from median imputation and multiple Bayesian imputation models. Each line represents a regression slope correlating the SC from each imputation method with predicted diagnostic outcomes, the average correlation of 0.98 highlights the consistency between imputation techniques.
