# Supplementary material for: Biological markers and psychosocial factors predict chronic pain conditions
Source: Nat Hum Behav. 2025 May 12;9(8):1710–25. doi: 10.1038/s41562-025-02156-y (PMC12367528; doi:10.1038/s41562-025-02156-y)
Supplement: Supplementary file 2 — Reporting Summary [file 41562_2025_2156_MOESM2_ESM.pdf]

nature portfolio

Corresponding author(s): Matt Fillingim & Etienne Vachon-Presseau

Last updated by author(s): Jan 2, 2025

Reporting Summary

Nature Portfolio wishes to improve the reproducibility of the work that we publish. This form provides structure for consistency and transparency in reporting. For further information on Nature Portfolio policies, see our Editorial Policies and the Editorial Policy Checklist.

Statistics

For all statistical analyses, confirm that the following items are present in the figure legend, table legend, main text, or Methods section.

n/a	Confirmed	
☐	☒	The exact sample size (*n*) for each experimental group/condition, given as a discrete number and unit of measurement
☐	☒	A statement on whether measurements were taken from distinct samples or whether the same sample was measured repeatedly
☐	☒	The statistical test(s) used AND whether they are one- or two-sided<br>*Only common tests should be described solely by name; describe more complex techniques in the Methods section.*
☐	☒	A description of all covariates tested
☐	☒	A description of any assumptions or corrections, such as tests of normality and adjustment for multiple comparisons
☐	☒	A full description of the statistical parameters including central tendency (e.g. means) or other basic estimates (e.g. regression coefficient) AND variation (e.g. standard deviation) or associated estimates of uncertainty (e.g. confidence intervals)
☐	☒	For null hypothesis testing, the test statistic (e.g. *F*, *t*, *r*) with confidence intervals, effect sizes, degrees of freedom and *P* value noted<br>*Give P values as exact values whenever suitable.*
☒	☐	For Bayesian analysis, information on the choice of priors and Markov chain Monte Carlo settings
☒	☐	For hierarchical and complex designs, identification of the appropriate level for tests and full reporting of outcomes
☐	☒	Estimates of effect sizes (e.g. Cohen's *d*, Pearson's *r*), indicating how they were calculated

Our web collection on statistics for biologists contains articles on many of the points above.

Software and code

Policy information about availability of computer code

Data collection	No software was used for data collection in this study. Data was sourced from three publicly available datasets: the UK Biobank, the All of Us Research Program (AoURP), and OpenPain repository. Details regarding data acquisition and collection methods for these datasets have been documented in previous work.
Data analysis	Data pre-processing and statistical analyses were performed using Python v.3.8 (including Numpy (v.1.22.0), Pandas (v.1.4.3), Scipy (v.1.10.1), Sklearn (v.1.3.2), Nilearn (v.0.10.0), Lifelines (v.0.26.4), Semopy (v.2.3.9), and SnapML (v.1.9.1)). Manuscript analysis code of the main figures will be available upon publication (https://github.com/EVPlab).

For manuscripts utilizing custom algorithms or software that are central to the research but not yet described in published literature, software must be made available to editors and reviewers. We strongly encourage code deposition in a community repository (e.g. GitHub). See the Nature Portfolio guidelines for submitting code & software for further information.

Data

Policy information about availability of data

All manuscripts must include a data availability statement. This statement should provide the following information, where applicable:

- Accession codes, unique identifiers, or web links for publicly available datasets
- A description of any restrictions on data availability
- For clinical datasets or third party data, please ensure that the statement adheres to our policy

All source data is available (upon data access application) from the UK Biobank. See https://www.ukbiobank.ac.uk/enable-your-research/apply-for-access for an application. The AoURP cohort can be obtained by registration: www.researchallofus.org. The OpenPain data is accessible through https://www.openpain.org/

Research involving human participants, their data, or biological material

Policy information about studies with human participants or human data. See also policy information about sex, gender (identity/presentation), and sexual orientation and race, ethnicity and racism.

Reporting on sex and gender	The sex of participants, as reported in the UK Biobank, was analyzed in relation to various biomarkers and psychosocial variables. We tested the generalizability of our models separately for males and females. Our analysis identified sex-specific effects in certain painful medical conditions associated with specific biomarkers. These findings are reported alongside other demographic variables, which are also presented separately. It is important to note that no information regarding gender identity was collected.
Reporting on race, ethnicity, or other socially relevant groupings	The study cohort consists of the general population from the United Kingdom, aged 40-70 years at the baseline visit, with 51-55% of participants being female and predominantly of white ethnicity (94-96%). After stratifying by painful medical conditions, the sample sizes were too small to estimate reliable effects to determine if models perform differently across ethnicities. For the replication cohorts (All of Us Research Program [AoURP] and OpenPain), demographics are more representative, and the models achieved comparable effect sizes to those in the UK Biobank. We report the age, ethnicity, and sex characteristics for each visit from each cohort.
Population characteristics	The study cohort consists of the general population from the United Kingdom, aged 40-70 years at the baseline visit, with 51-55% of participants being female and predominantly of white ethnicity (94-96%). Participants provided electronic health records that detail current and past clinical diagnoses or provided self-reported medical history. In all machine learning models, age and sex were controlled to reliably estimate the biological effects stemming from the disease process.
Recruitment	*Describe how participants were recruited. Outline any potential self-selection bias or other biases that may be present and how these are likely to impact results.*
Ethics oversight	All participants provided written, informed consent, and the study was approved by the Research Ethics Committee (REC number 11/NW/0382). Further information on the consent procedure can be found elsewhere (https://biobank.ndph.ox.ac.uk/ukb/field.cgi?id=200).

Note that full information on the approval of the study protocol must also be provided in the manuscript.

Field-specific reporting

Please select the one below that is the best fit for your research. If you are not sure, read the appropriate sections before making your selection.

☒ Life sciences ☐ Behavioural & social sciences ☐ Ecological, evolutionary & environmental sciences

For a reference copy of the document with all sections, see nature.com/documents/nr-reporting-summary-flat.pdf

Life sciences study design

All studies must disclose on these points even when the disclosure is negative.

Sample size	No sample-size calculation was done. A total of 493,211 participants were included in this study, using the UK Biobank. For the replication cohorts, the AoURP had a total sample size of 27,151 and OpenPain had a total sample of 250 participants.
Data exclusions	In the UK Biobank, participants with more than 50% of missing features within each biological and psychosocial modality were excluded. No other exclusion criteria were applied to ensure that the study findings would be as generalizable as possible to the wider population. For the All of Us Research Program (AoURP), participants were excluded if they had missing assays from the routine composite blood panel or if they lacked diagnoses of any of the 13 conditions included in the composite blood assay signature. A healthy control group was created by selecting participants with no clinical records of inflammatory, musculoskeletal, or cardiovascular diagnoses. In the OpenPain project, participants were excluded if they had corrupt imaging data or if they did not complete the pain questionnaire.
Replication	All models were derived and validated within the UK Biobank cohort using nested cross-validation to ensure robustness. For select biomarkers, exact replications were conducted in additional cohorts: the All of Us Research Program (AoURP) for the blood-based biomarker (n=27,151) and OpenPain for the brain imaging biomarker (n=250).

Randomization | The study design did not include randomization of participants.

Blinding | Blinding was not necessary for this study, as the determination of feature importance was conducted algorithmically through the machine learning models. For the routine panel replication in the All of Us Research Program, we selected the top 10 assays based on their feature importance as assigned by the model developed using the UK Biobank data.

Reporting for specific materials, systems and methods

We require information from authors about some types of materials, experimental systems and methods used in many studies. Here, indicate whether each material, system or method listed is relevant to your study. If you are not sure if a list item applies to your research, read the appropriate section before selecting a response.

Materials & experimental systems

n/a	Involved in the study
☒	☐ Antibodies
☒	☐ Eukaryotic cell lines
☒	☐ Palaeontology and archaeology
☒	☐ Animals and other organisms
☒	☐ Clinical data
☒	☐ Dual use research of concern
☒	☐ Plants

Methods

n/a	Involved in the study
☒	☐ ChIP-seq
☒	☐ Flow cytometry
☐	☒ MRI-based neuroimaging

Plants

Seed stocks | *Report on the source of all seed stocks or other plant material used. If applicable, state the seed stock centre and catalogue number. If plant specimens were collected from the field, describe the collection location, date and sampling procedures.*

Novel plant genotypes | *Describe the methods by which all novel plant genotypes were produced. This includes those generated by transgenic approaches, gene editing, chemical/radiation-based mutagenesis and hybridization. For transgenic lines, describe the transformation method, the number of independent lines analyzed and the generation upon which experiments were performed. For gene-edited lines, describe the editor used, the endogenous sequence targeted for editing, the targeting guide RNA sequence (if applicable) and how the editor was applied.*

Authentication | *Describe any authentication procedures for each seed stock used or novel genotype generated. Describe any experiments used to assess the effect of a mutation and, where applicable, how potential secondary effects (e.g. second site T-DNA insertions, mosiacism, off-target gene editing) were examined.*

Magnetic resonance imaging

Experimental design

Design type | UK Biobank brain imaging resting-state functional MRI scans

Design specifications | Single 6-minutes resting-state run, eyes open. T1 susceptibility-weighted structural imaging. Diffusion weighted imaging.

Behavioral performance measures | The number of self-reported pain sites, specific pain body sites, and 35 distinct pain-associated medical conditions.

Acquisition

Imaging type(s) | UK Biobank brain imaging data: structural (T1 susceptibility-weighted), diffusion weighted, and resting-state functional scans - See Methods for details.

Field strength | 3T

Sequence & imaging parameters | Please see Miller et al., Nature Neuroscience 2016 for a full list of the imaging parameters.

Area of acquisition | Whole brain

Diffusion MRI ☒ Used ☐ Not used

Parameters | 100 distinct diffusion-encoding directions, 50x b=1000 s/mm2, 50x b=2000 s/mm2, multi-shell, no cardiac gating

Preprocessing

Preprocessing software	fsl, nilearn, AFNI
Normalization	Minimal processing was done according to Miller et al., Nature Neuroscience 2016. Additional processing was conducted including despiking (AFNI from Nipype), 6-mm kernel smoothing (Nilearn), and resampling to 3-mm (for storage purposes) to resemble an a-priori brain-based signature for sustained pain (ToPS; see Lee et al., 2021 Nature Medicine).<br><br>The preprocessing of diffusion imaging data from the UK Biobank involved using Eddy and BEDPOSTx outputs that remained in the space and resolution of the native diffusion data space after gradient distortion correction (GDC). A nonlinear transformation, as estimated by Tract-Based Spatial Statistics (TBSS), was applied to align this data into the 1mm MNI standard space for generating tractography results in this standardized space.
Normalization template	Data were normalize to MNI152 template space.
Noise and artifact removal	Minimal processing was done according to Miller et al., Nature Neuroscience 2016. MRI-based covariates included head motion (linear, squared, and cubed), imaging site, position in the scanner, and coil position (Z, Y, Z respectively). Two deconfounding framework were used - see Method for details.<br><br>The preprocessing of the diffusion imaging data begins with correction for eddy currents and head motion using the Eddy tool, which also addresses outlier slices in the 4D data set. Following this, gradient distortion correction (GDC) is applied, culminating in the production of the 4D output file. This process is detailed in the documentation found at FSL's Eddy tool website and is based on methodologies described by Andersson and Sotiropoulos in their 2015 and 2016 publications.
Volume censoring	N/A

Statistical modeling & inference

Model type and settings	Logistic regression machine learning models were trained using a nested 5-fold cross-validation framework to classify participants either reporting chronic pain or diagnosed with pain-associated medical conditions from their pain-free or diagnosis-free counterparts. These models utilized imaging features derived from three types of scans: resting-state fMRI, diffusion-weighted tractography, and T1 susceptibility-weighted anatomical imaging. The performance of the models was evaluated on the left-out subjects from the testing folds, and quantified using the receiver operating characteristic area under the curve (ROC-AUC) to measure the accuracy of the classification.
Effect(s) tested	Effects of chronic pain phenotypes and pain-associated diagnoses on various brain-based imaging derived phenotypes - see Methods for details.

Specify type of analysis: ☒ Whole brain ☐ ROI-based ☐ Both

Statistic type for inference<br>(See Eklund et al. 2016)	This study used functional connectivity, anatomical integrity (e.g., cortical thickness, gray matter volume), and white matter tractography (e.g., fractional anisotropy, mean diffusivity).
Correction	Significance of group comparisons was determined using false discovery rate (q = 0.05).

Models & analysis

n/a	Involved in the study
☐	☒ Functional and/or effective connectivity
☒	☐ Graph analysis
☐	☒ Multivariate modeling or predictive analysis

Functional and/or effective connectivity	DCC was used for Dynamic Connectivity following the same signature extraction from the Tonic Pain Signature (see Lee et al., 2021 Nature Medicine).
Multivariate modeling and predictive analysis	independent variables include all imaging derive phenotypes. No feature extraction or dimension reduction was used. The logistic regression ridge (l2 penalty) was tuned using randomized hyperameter search to optimize feature weights. ROC-AUC scores were used to optimize and evaluate models.
